# Pulmonary lobar torsion: a rare complication following pulmonary resection, but one not to miss

**DOI:** 10.1259/bjrcr.20160010

**Published:** 2016-07-01

**Authors:** Lucy Childs, Steve Ellis, Olivia Francies

**Affiliations:** Royal London Hospital, Barts Health NHS Trust, London, UK

## Abstract

Lobar torsion is an uncommon phenomenon but a crucial diagnosis to consider in any patient undergoing lobectomy, as the clinical findings and radiographic appearances are non-specific. This case report documents the clinical and radiological evolution of middle lobe torsion in a patient who underwent right upper lobectomy for Stage 1 adenocarcinoma of the lung. The diagnosis of lobar torsion is most often made on CT scanning of the chest, which is frequently performed in order to distinguish this from multiple other more frequently encountered post-operative complications. Contrast-enhanced CT scan is the recommended imaging modality in suspected cases. If features of lobar torsion are identified, the findings must be communicated immediately to cardiothoracic surgeons owing to the potentially life-threatening consequences of delay. Management of lobar torsion is predominantly surgical, with several techniques currently in use; however, video-assisted thoracoscopic surgery is emerging as an increasingly favoured approach.

## Clinical presentation

**Figure 1.**
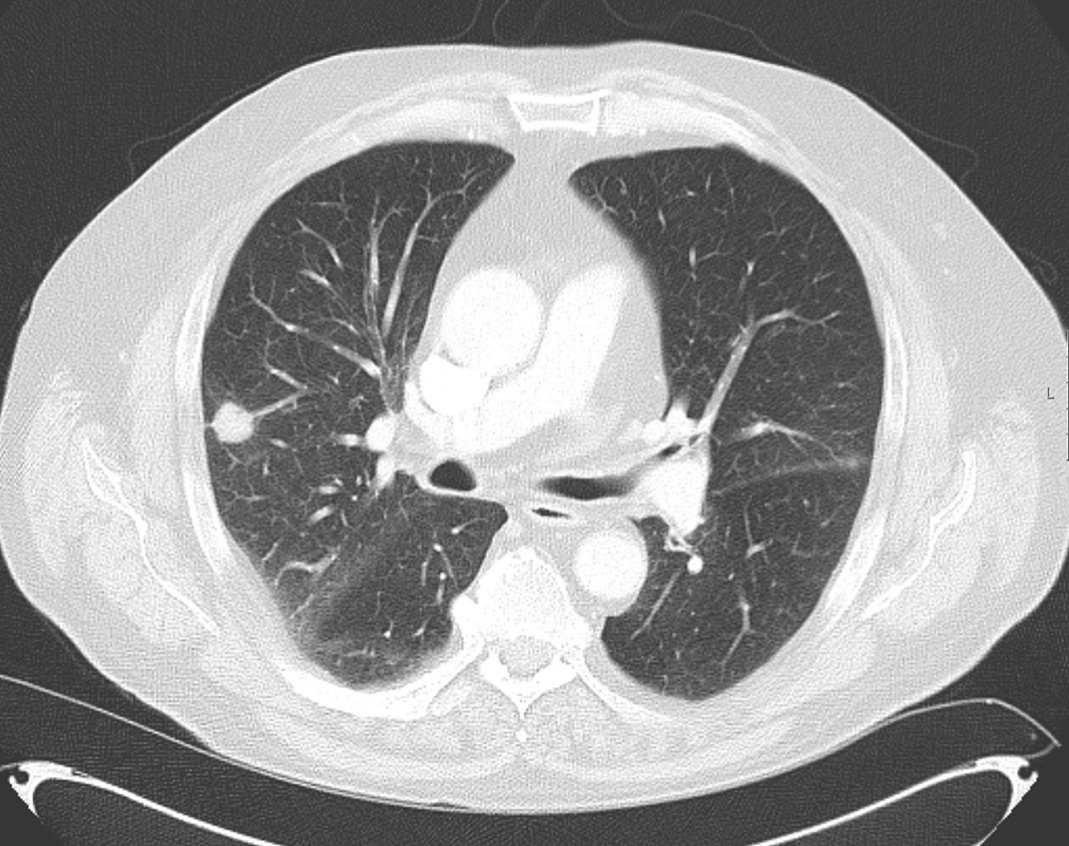
Axial lung window reformat of original diagnostic CT image demonstrating a small soft tissue nodule in the right upper lobe.

This text only for test. An 80-year-old male patient with persistent cough and an abnormal chest X-ray was referred to the respiratory physicians. The patient had a long history of smoking and an established diagnosis of chronic obstructive pulmonary disease. Contrast-enhanced CT scan of the chest was performed as part of the diagnostic work-up and demonstrated a small 12 mm soft tissue nodule in the right upper lobe (Figure 1). The nodule exhibited intense tracer uptake on positron emission tomography scanning, with no mediastinal nodal uptake to suggest nodal metastatic disease. After discussion in the multidisciplinary team meeting, the patient was referred to the cardiothoracic surgeons for video-assisted thoracoscopic surgery (VATS) right upper lobectomy and mediastinal lymph node resection.

### Post-operative period

**Figure 2.**
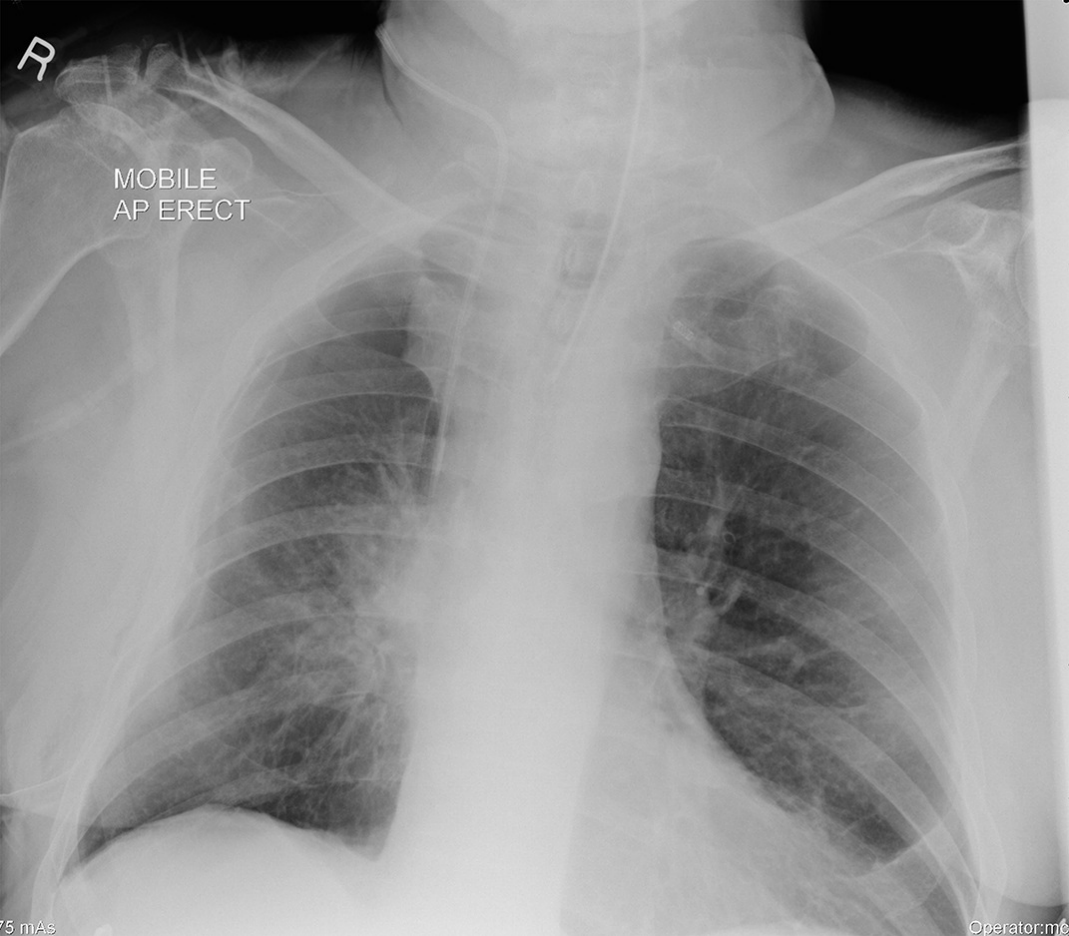
The first post-operative chest radiograph performed within hours of upper lobectomy. There is increased density adjacent to the right hilum but no lobar parenchymal consolidation. A right central venous catheter, endotracheal tube and right chest drain are also demonstrated. AP, anteroposterior.

**Figure 3.**
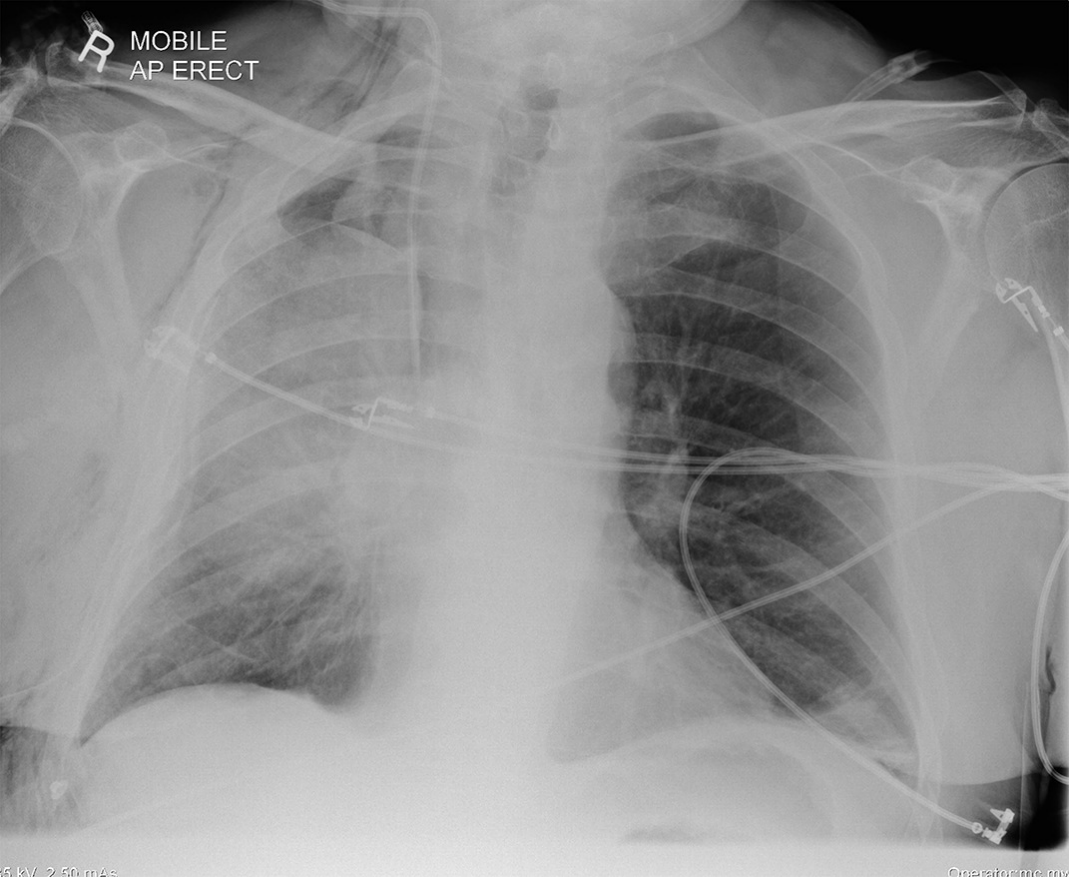
Chest radiograph performed 48 h postoperatively. There has been an increase in the right perihilar opacification, with well-defined superior and inferior margins formed by the boundaries of the torted right middle lobe; the diagnosis could have been suspected at this point. Surgical emphysema is seen throughout the soft tissues of the right chest wall. AP, anteroposterior.

**Figure 4.**
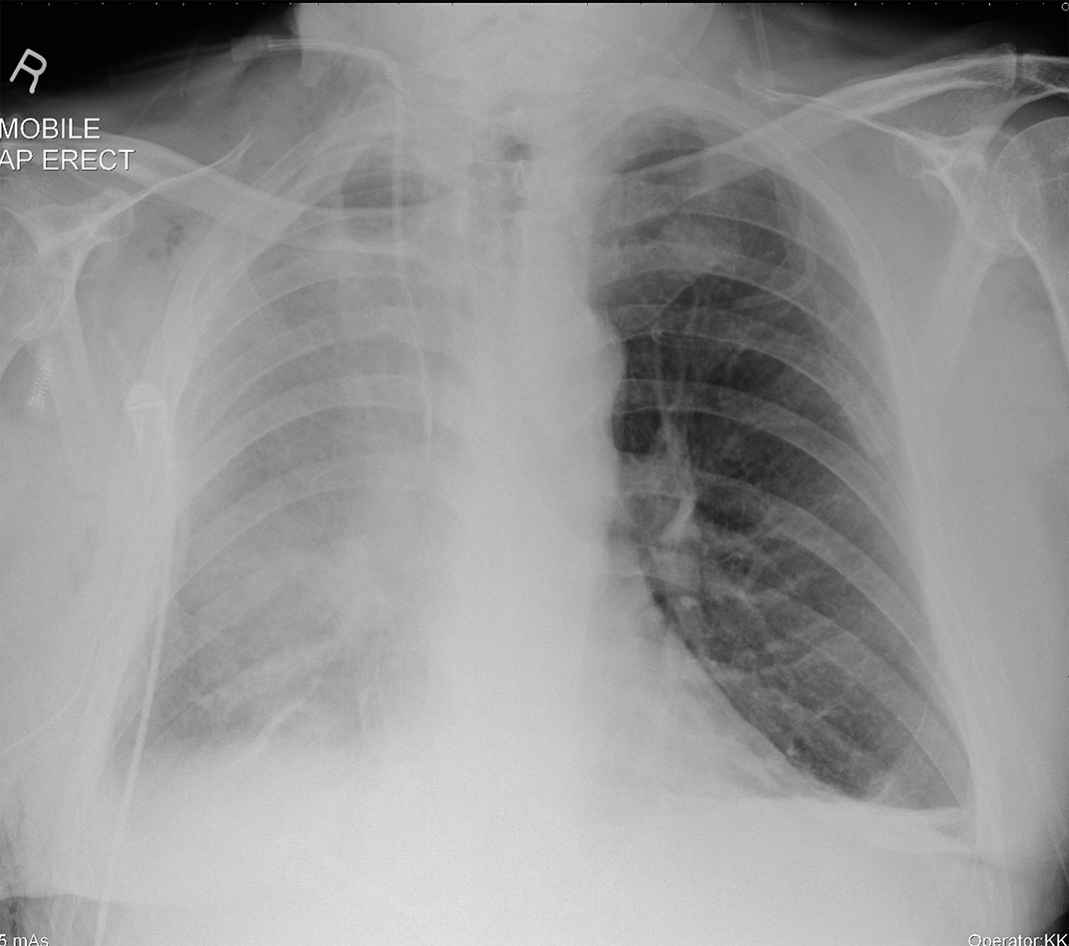
Chest radiograph on post-operative day 3 confirms further deterioration, with more extensive opacification projected over most of the hemithorax but sparing the costophrenic angle and apex. AP, anteroposterior.

The immediate post-operative period was uneventful. However, within 24 h, the patient became progressively hypoxic. A chest X-ray performed 24 h after surgery demonstrated significant deterioration in the radiological appearance, with diffuse consolidation in the right upper and mid zones (Figure 2). Over the subsequent 48 h, the radiographic appearances progressively worsened (Figures 3 and 4). Treatment with non-invasive ventilation and intensive physiotherapy did not alter the deteriorating clinical course of rising white cell count, development of fever and persistent hypoxia. A CT scan was performed on post-operative day 3 for further evaluation.

## Imaging finding

**Figure 5.**
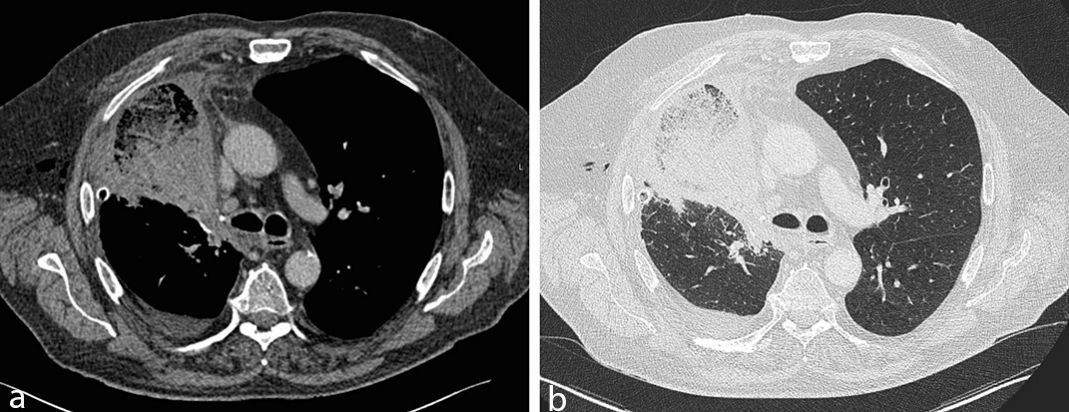
Axial images from the post-operative contrast-enhanced CT scan. (a) Soft tissue window shows an expanded, poorly enhancing right middle lobe, which tapers towards the hilum. (b) Lung window demonstrates extensive ground-glass opacity, consolidation and interlobular septal thickening.

A series of three chest X-rays performed on consecutive days post lobectomy demonstrated increasing opacification, initially perihilar but subsequently projected over the majority of the right hemithorax, sparing only the costophrenic angle and apex. On the 48-h post-lobectomy chest radiograph, there were well-defined superior and inferior margins to the opacity, reflecting the borders of the torted right middle lobe, which may have been suggestive of the final diagnosis at that time. Contrast-enhanced CT scan of the chest demonstrated a superiorly located and expanded right middle lobe with diffuse ground-glass consolidation and interlobular septal thickening. The middle lobe parenchyma lacked enhancement and the bronchovascular pedicle at the right hilum tapered into the abnormal middle lobe (Figures 5a,b 6a,b, and 7). A diagnosis of middle lobe torsion was suspected based on the CT scan appearances and this was immediately communicated to the cardiothoracic surgeons who performed a VATS right middle lobectomy after confirmation of intraoperative lobar torsion. After further treatment with antibiotics for post-operative pneumonia and rigid bronchoscopy with aspiration of thick secretions, the patient recovered and was finally discharged home with outpatient follow-up.

**Figure 6.**
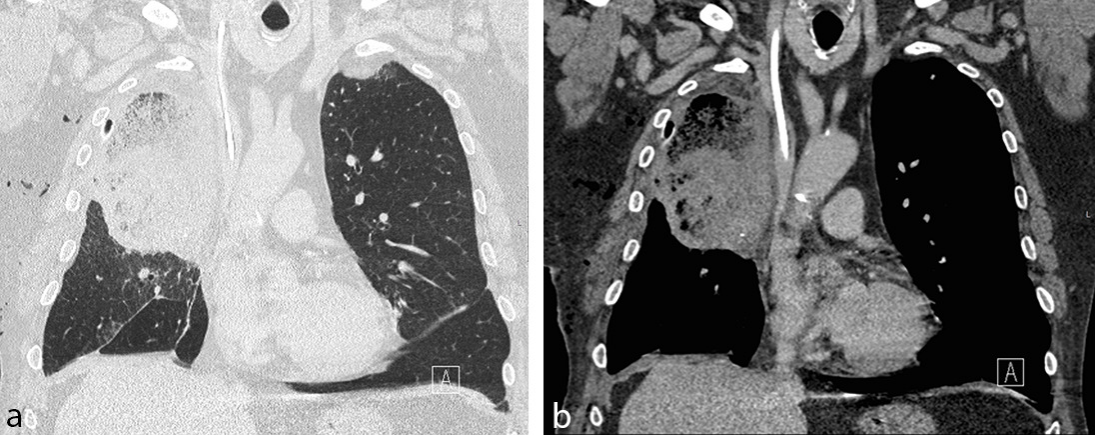
Coronal reformat of the post-operative contrast-enhanced CT scan. (a) Lung window setting. The middle lobe is expanded and superiorly located with extensive ground-glass consolidation. (b) Soft tissue window setting. The middle lobe is expanded and superiorly located with extensive ground-glass consolidation.

**Figure 7.**
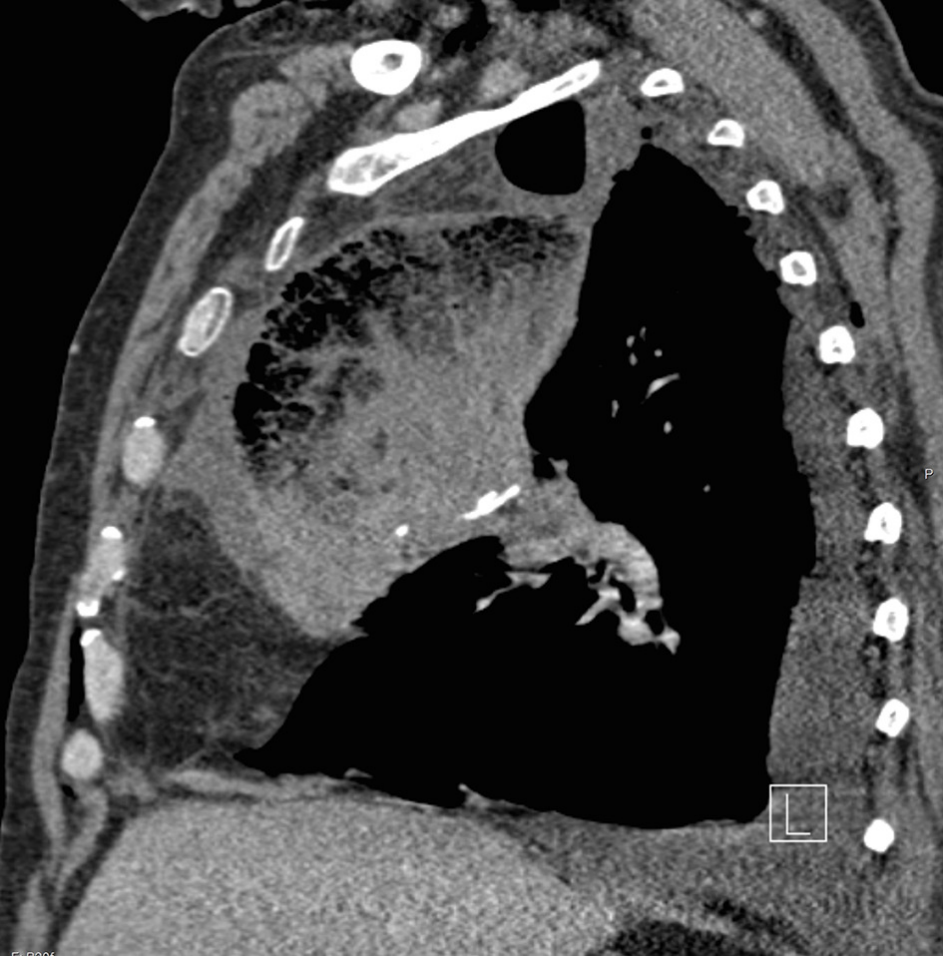
Sagittal reformat of the post-operative contrast-enhanced CT scan on soft tissue window. The middle lobe can be seen tapering towards the hilum and hilar surgical clips. There is interlobular septal thickening and expansion of the poorly enhancing superiorly positioned middle lobe.

Histopathological analysis of the resected middle lobe demonstrated haemorrhagic necrosis, which was consistent with infarction. The right upper lobe resection specimen confirmed adenocarcinoma (pT1a,pN0,pM0).

## Differential diagnosis

The main differential diagnoses include infection, postoperative lobar collapse, pulmonary oedema, aspiration pneumonia and pulmonary contusions; the absence of enhancement would not fit with these alternatives. Of note, necrotising pneumonia may have similar imaging findings with expansion of the lobe and associated consolidation, and perhaps lack of enhancement, but tapering of the bronchovascular bundle does not fit any of these differentials.

## Discussion

Lobar torsion is rare, occurring in 0.089–0.3% of patients following lobectomy.^1^ The complication is most commonly observed after thoracic surgery, with torsion of the middle lobe following right upper lobectomy, the most common pre-emptive surgical procedure. Lobar torsion may also occur in the context of traumatic thoracic injury, usually in cases of pneumothorax. Torsion may also occur spontaneously, often with an underlying, associated abnormality such as diaphragmatic hernia or bronchial carcinoid.^2,3^ Predispositions to torsion include heavily consolidated airless lobes, long hilar pedicle, a complete oblique fissure (an incomplete interlobular fissure tethers the lobe and prevents rotation), large pneumothoraces, large pleural effusions and transection of the inferior pulmonary ligament, which is usually postoperative or traumatic.

Symptoms and signs often have an abrupt, but non-specific, clinical onset, presenting soon after surgery with cough and breathlessness accompanied by tachypnoea, tachycardia, hypoxia and fever.^1^ Radiological findings also evolve rapidly. X-ray findings include a collapsed or consolidated lung, which may be observed in an unusual orientation to that expected.^3^ On CT scan, the lobe will typically enhance poorly and have either ground-glass attenuation or undergo complete collapse. The bronchi and hilar vessels supplying the lobar torsion exhibit a cut-off in the absence of a distally obstructing mass.^4,5^ Displacement of the hilum due to abnormal rotation and lobar air trapping with bulging and expansion of the twisted lobe owing to haemorrhagic engorgement and infarction may also be observed.

Mortality is high if the complication goes unrecognised, or if urgent surgical fixation or further lobectomy is not performed. Thoracotomy and resection of the non-viable lobe has traditionally been the typical management. More recently, VATS lobectomy has emerged as an increasingly successful approach in the management of this rare complication.^6,7^ Intraoperative techniques to fix the middle lobe in place include suturing the lung to the parietal pleura, clipping the middle and lower lobes together, synthetic polymeric sealants and use of fibrin glue to appose the surfaces.^3,8,9^

## Learning points

1. Lobar torsion is a rare and potentially life-threatening pulmonary pathology, most commonly occurring after thoracic surgery.
2. Lobar torsion usually presents with non-specific clinical signs of tachypnoea, tachycardia and fever, and therefore requires a high index of suspicion.
3. Radiographic features include rapid onset of lobar collapse or consolidation, lobar expansion and an unusual position of the consolidated lobe.
4. Mortality is high if lobar torsion goes unrecognised.
5. Treatment with thoracotomy or VATS lobectomy is the usual treatment of choice; prophylactic fixation is often performed to prevent this complication.

## Consent

Written informed consent was obtained from the patient’s wife for publication of this case report, including accompanying images. Sadly, the patient himself is now deceased.

## References

[1] Cable DG, Deschamps C, Allen MS, Miller DL, Nichols FC, Trastek VF, et al Lobar torsion after pulmonary resection: presentation and outcome. J Thorac Cardiovasc Surg 2001; 122: 1091–3. 11726883 10.1067/mtc.2001.117839

[2] Berkmen YM, Yankelevitz D, Davis SD, Zanzonico P Torsion of the upper lobe in pneumothorax. Radiology 1989; 173: 447–9. 2798876 10.1148/radiology.173.2.2798876

[3] Felson B Lung torsion: radiographic findings in nine cases. Radiology 1987; 162: 631–8. 3809475 10.1148/radiology.162.3.3809475

[4] Kanaan S, Boswell WD, Hagen JA Clinical and radiographic signs lead to early detection of lobar torsion and subsequent successful intervention. J Thorac Cardiovasc Surg 2006; 132: 720–1. 16935149 10.1016/j.jtcvs.2006.05.028

[5] Kim EA, Lee KS, Shim YM, Kim J, Kim K, Kim TS, et al Radiographic and CT findings in complications following pulmonary resection. Radiographics 2002; 22: 67–86. 11796900 10.1148/radiographics.22.1.g02ja0367

[6] Sticco CC, Andaz S, Fox S Middle lobe torsion after right upper lobectomy: a report of video-assisted thoracoscopic management. J Thorac Cardiovasc Surg 2007; 134: 1090–1. 17903554 10.1016/j.jtcvs.2007.05.046

[7] McKenna RJ, Houck W, Fuller CB Video-assisted thoracic surgery lobectomy: experience with 1,100 cases. Ann Thorac Surg 2006; 81: 421–5. 16427825 10.1016/j.athoracsur.2005.07.078

[8] Venuta F, Anile M, de Giacomo T, Coloni GF Prevention of middle lobe torsion after right upper lobectomy with a polymeric sealant. J Thorac Cardiovasc Surg 2012; 143: 240–1. 21798565 10.1016/j.jtcvs.2011.06.033

[9] Kutlu CA, Olgac G Pleural flap to prevent lobar torsion: a novel technique. Eur J Cardiothorac Surg 2006; 30: 943–4. 17052913 10.1016/j.ejcts.2006.09.015

